# Antimicrobial Resistance Profiles of Clinically Relevant Enterobacteriaceae Isolates: A Retrospective Study at a Southern Italian Hospital

**DOI:** 10.3390/antibiotics14090899

**Published:** 2025-09-05

**Authors:** Giorgia Stornelli, Beatrice Marinacci, Valentina Puca, Benedetta Pellegrini, Roberta Zita Marulli, Ivana Cataldo, Francesca Sisto, Vittoria Perrotti, Arianna Pompilio, Mattia Mori, Pamela Di Giovanni, Rossella Grande

**Affiliations:** 1 Department of Pharmacy, “G. d’Annunzio” University of Chieti-Pescara, 66100 Chieti, Italy; giorgia.stornelli@phd.unich.it (G.S.); beatrice.marinacci@unich.it (B.M.); valentina.puca@unich.it (V.P.); benedetta.pellegrini@unich.it (B.P.); 2 Department of Innovative Technologies in Medicine & Dentistry, University of Chieti-Pescara, 66100 Chieti, Italy; vittoria.perrotti@unich.it; 3 Operative Unit of Clinical Pathology, S. Pio Hospital, 66054 Chieti, Italy; rzita.marulli@asl2abruzzo.it (R.Z.M.); ivana.cataldo@asl2abruzzo.it (I.C.); 4 Department of Biomedical, Surgical and Dentistry Sciences, University of Milano, 20133 Milano, Italy; francesca.sisto@unimi.it; 5 Department of Medical, Oral and Biotechnological Sciences, “G. d’Annunzio” University of Chieti-Pescara, 66100 Chieti, Italy; arianna.pompilio@unich.it; 6 Department of Biotechnology, Chemistry and Pharmacy, University of Siena, Via Aldo Moro 2, 53100 Siena, Italy; mattia.mori@unisi.it; 7 Department of Medicine and Ageing Sciences, “G. d’Annunzio” University of Chieti-Pescara, 66100 Chieti, Italy; pamela.digiovanni@unich.it

**Keywords:** Enterobacteriaceae, antimicrobial resistance, nosocomial infections, *Escherichia coli*, *Klebsiella pneumoniae*, *Proteus mirabilis*, extended-spectrum beta-lactamase, carbapenemase-producing bacteria, retrospective study

## Abstract

Background: The Enterobacteriaceae family is the most heterogeneous group of Gram-negative bacilli, with both environmental and clinical relevance. Although many of these species are part of the normal intestinal microbiota, species such as *Escherichia coli*, *Klebsiella pneumoniae*, and *Proteus mirabilis* are among the most common opportunistic pathogens, frequently responsible for nosocomial infections, including urinary tract infections, bacteraemia, and pneumonia. Based on these concerns, these species are increasingly being studied for their ability to develop antimicrobial resistance, often mediated by extended spectrum β-lactamase or carbapenemase production. The present study aims to analyse the antimicrobial resistance profiles of Enterobacteriaceae isolated from a southern Italian hospital focusing on the species of major clinical importance. Methods: A retrospective analysis was carried out on biological samples collected between 2019 and 2023 at the Microbiology Laboratory of the San Pio Hospital, Vasto (Italy). Eight hundred ninety patients were included, with an average age of 73 years. Bacterial identification was carried out using bacterial culture and biochemical methods, while antimicrobial sensitivity was assessed by using the automated Walk Away System. Results: The most frequently isolated species were *Escherichia coli* (63.2%), *Klebsiella* spp. (21.9%), and *Proteus* spp. (8.8%). All isolates showed resistance to at least one antimicrobial and most to more than four. The highest resistance rates were observed for Cefotaxime (16.0%), followed by Ampicillin (15.6%) and Ciprofloxacin (13.2%). Conclusions: The high prevalence of antimicrobial resistance among clinically relevant Enterobacteriaceae species is a growing clinical challenge. The results of this study, which describe the trend of resistance among Enterobacteriaceae in a local hospital, could help to raise awareness highlighting the urgent need for more advanced diagnostic tools and new therapeutic strategies to treat infections caused by multi-resistant microorganisms.

## 1. Introduction

In recent decades, the increasing spread of antimicrobial resistance (AMR) has become one of the most significant challenges for modern medicine, threatening the efficacy of treatments for a wide range of bacterial infections [1]. The scientific community has risen to the challenge, trying to find alternative strategies to combat AMR: innovative targets [2,3], probiotics-derived metabolites [4], phage therapy [5], and genetically modified strains [6,7] are few examples of future alternative approaches [8,9]. According to the updated Bacterial Priority Pathogens List (BPPL) by the World Health Organization (WHO), which highlights the most critical antibiotic-resistant bacteria to inform global health strategies, investments and research should prioritise the Enterobacteriaceae family [10]. The Enterobacteriaceae are a family of Gram-negative rod-shaped bacteria that constitute a natural component of the human intestinal microbiota [11]. However, these microorganisms are also among the most common human opportunistic pathogens, responsible for a wide range of infections such as cystitis, pyelonephritis, sepsis, and pneumonia, as well as peritonitis, meningitis, and medical device-associated infections [12]. These bacteria represent one of the leading causes of both community- and hospital-acquired infections, with *Escherichia coli* (*E. coli*) emerging as by far the most frequently isolated pathogen [13,14,15].

The synthesis of β-lactamase enzymes is one of the main causes of antibiotic resistance in Gram-negative bacteria [16]. Although three main molecular mechanisms, such as enzyme production, efflux pump overexpression, and porin modification, are involved in the development of resistance to β-lactamic-antibiotics, in Enterobacteriaceae and other Gram-negative bacteria, enzymatic inactivation is the predominant mechanism [17], and either alone or in combination with the others, it is associated with the multi-resistant phenotypes observed in clinical isolates [18,19].

Bacterial β-lactamase enzymes hydrolyse the β-lactam ring, making it inactive. Extended-spectrum β-lactamases (ESBLs) are able to hydrolyse and confer resistance to a variety of newer β-lactam antibiotics, including monobactams such as Aztreonam and extended-spectrum cephalosporins, also known as third-generation cephalosporins, such as Cefotaxime, Ceftriaxone, and Ceftazidime [20]. Cephamycins (e.g., Cefoxitin and Cefotetan) and carbapenems (e.g., Imipenem, Meropenem, and Ertapenem), however, are unaffected by these enzymes. Although similar enzymes have also been identified in various other members of the Enterobacteriaceae family and in several non-fermenting bacteria, *Klebsiella pneumoniae* (*K. pneumoniae*) and *E. coli* continue to be the principal ESBLs-producing organisms in the world [21,22]. ESBLs and carbapenemases producing Enterobacteriaceae (CPE) are attracting significant clinical attention [23], because of the increased mortality, delayed effective therapy, prolonged hospitalisation, and increased healthcare expenditures [24].

For many years, β-lactam antimicrobials such as Meropenem have been the most effective therapeutic agents for treating severe infections caused by Gram-negative bacteria [25]. However, as reported in the literature, the development and spread of β-lactamase resistance mechanisms in Gram-negative bacilli have significantly reduced the efficacy of earlier β-lactam agents requiring the development of new more potent extended-spectrum agents [26,27]. Genes encoding most β-lactamases have been identified on plasmids or transposons that can be easily transferred to other strains and often carry additional genetic elements conferring resistance to different antimicrobial classes. The most prevalent ESBLs worldwide are the CTX-M type enzymes, which are found both in the community and hospitals [28]. Another mechanism contributing to increased β-lactam resistance is the production of plasmid-mediated AmpC enzymes, which have also been detected in commonly isolated bacteria such as *E. coli* and *Klebsiella* spp. [29].

The spread of AMR, particularly among Gram-negative bacteria, represents an increasingly serious concern in modern medicine worldwide, although the identified pathogens and resistance mechanisms exhibit geographical variability [30]. The dissemination of CPE is particularly alarming in Italy: one-third of *K. pneumoniae* strains isolated from blood or cerebrospinal fluid (CSF), are resistant to carbapenems. This phenomenon has been described as an endemic situation caused by carbapenemase (KPC) producing *K. pneumoniae* and as an inter-regional spread in the case of strains producing Verona integron-encoded metallo-β-lactamase (VIM) [31].

The aim of this study is to carry out a retrospective analysis of bacterial resistance profiles against antimicrobials commonly used in clinical practice, with a specific focus on the Enterobacteriaceae family. In our research, we considered only the samples collected from an Italian hospital located in Abruzzo (Vasto) obtained over a five-year period. The analysis allowed us to identify the main resistance patterns associated with this group of bacteria, highlighting the presence of multi-resistance. The results provide a useful overview for optimising infection management in clinical settings, supporting clinicians in selecting more targeted and effective therapies, and helping to reduce the use of antibiotics that are no longer suitable for the treatment of Enterobacteriaceae infections. Furthermore, this study contributes to the adaptation of therapeutic guidelines to the specificities of the local epidemiological context, which may differ significantly from that of other geographical areas.

## 2. Results

### 2.1. Distribution of Samples

The distribution of the 890 samples that were evaluated throughout the 2019–2023 period varied by year: 193 samples (21.7%) were collected in 2019, 119 samples (13.4%) in 2020, 178 samples (20.0%) in 2021, 182 samples (20.4%) in 2022, and 218 samples (24.5%) in 2023. The gender distribution revealed a predominance of female patients, with 61.3% (n = 546) females and 38.7% (n = 344) males. Among them, 58.5% (n = 520) were hospitalised, while the remaining 41.5% (n = 370) were non-hospitalised. In terms of co-infections, 93.2% (n = 830) of the samples did not show co-infection, whereas only 6.8% (n = 60) presented co-infections (Table 1).

The 890 samples were predominantly obtained from patients hospitalised in wards such as Infectious Diseases (6.4%, n = 56), Internal Medicine (9.3%, n = 83), and Geriatrics (9.3%, n = 83). Less than 1% of the samples were collected from Cardiology, Gynaecology, Obstetrics, and Psychiatry, whereas wards such as Surgery (2.6%, n = 23), Neurology (2.0%, n = 18), and Cardiac Intensive Care (1.0%, n = 9) did not show a notable percentage of samples collected. The samples from the non-hospitalised patients were obtained at Community Health Centres (18.4%, n = 164), outpatient facilities (32.4%, n = 288), and home assistance (7.5%, n = 67). In addition, a small percentage of samples were collected from correctional facilities (0.1%, n = 1) (Table 2).

As for the types of specimens collected (Table 3), the most frequently obtained specimens were from urine, representing 79.3% (n = 705) of the total samples, followed by blood (8.4%, n = 75) and skin swabs (4.0%, n = 36). Other specimens included vaginal swabs (2.5%, n = 22), sputum (1.6%, n = 14), and peritoneal fluid (1.5%, n = 13). Less common specimens included tracheal aspirates (0.9%, n = 8), broncho-alveolar lavage (0.2%, n = 2), and joint fluid (0.1%, n = 1). A variety of swabs (rectal, cervical, nasal, and umbilical) and fluids (seminal, pus) represented less than 1% of the samples, as shown in Table 3.

### 2.2. Distribution of Isolated Enterobacteriaceae

The microbiological analysis revealed a diverse spectrum of microorganisms that have been grouped according to the species level as shown in Table 4. When “spp.” is reported, at least 2 or more distinguished species were identified. Because 60 samples presented co-infections, in the same sample, more than one microorganism was found. This is the reason why the percentage was calculated on the total number of microorganisms isolated (n = 956). *E. coli* was the most prevalent microorganism, identified in 63.2% (n = 604) of the samples, followed by *Klebsiella* spp. (21.9%, n = 209) and *Proteus* spp. (8.8%, n = 84). Other common pathogens included *Enterobacter* spp. (2.1%, n = 20) and *Morganella morganii* (*M. morganii*) (1.8%, n = 17). Less frequently encountered species were *Citrobacter* spp. (1.2%, n = 12), and *Serratia marcescens* (*S. marcescens*) (0.8%, n = 8). *Acinetobacter baumannii* (*A. baumannii*) is not part of the Enterobacteriaceae family; however, data regarding this pathogen were included, because it was detected, even though at a minor percentage (0.1%, n = 1) (Table 4).

### 2.3. Distribution of Multi-Resistance

The distribution of resistance versus different antimicrobials, regardless of the antimicrobial class, indicated as multi-resistance, was analysed according to age, by dividing the population into four groups: ≤65 years, 66–75 years, 76–85 years, and >85 years. The percentage of patients infected by multi-resistant bacteria was homogeneous in all categories, ranging from 97.7% in the ≤65 age group to 99.0% in that over 85 years (Table 5). Overall, out of a total of 890 patients, 876 were infected by multi-resistant strains (98.4%), and only 14 patients (1.6%) were not (Table 8).

The distribution of multi-resistance was evaluated not only by age distribution but also by gender (Table 6). Among a total of 890 patients, 61.3% were female, and 38.7% were male (Table 1). Among the subjects who did not show multi-resistance, the distribution between the two genders was equal (50.0% for both), while among those with multi-resistance, 61.5% were female and 38.5% were male. Overall, multi-resistance appeared slightly more frequent in female patients (61.5%).

By analysing the frequency of multi-resistance in relation to the year of sampling, it appeared homogeneous across years, with a slight prevalence in 2023 (24.8%) and the lowest value in 2020 (13.1%) which is the only one that does not properly fit in the observed trend. The percentage of patients without multi-resistance also showed a time-varying trend, ranging from 7.1% in 2023 to 14.3% in 2019 (Table 7).

**Figure 1:**
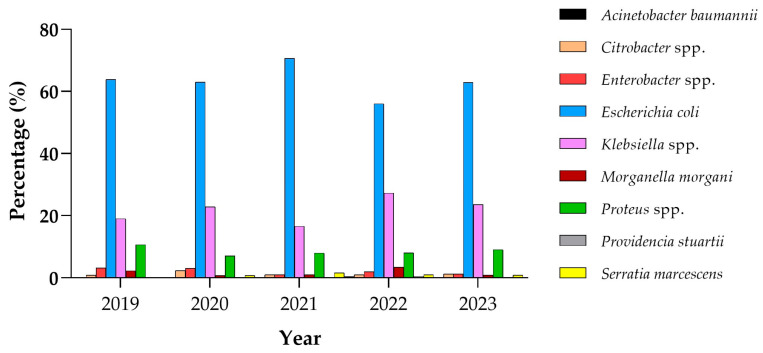
Percentage of microorganisms isolated per year.

As shown in Figure 1, *E. coli* was the most identified pathogen across all years, representing 63.8% of total samples in 2019, 63% in 2020, 70.6% in 2021, 56.1% in 2022, and 62.9% in 2023. This indicates that *E. coli* remained the dominant microorganism, although its prevalence fluctuated over the years. A similar trend was observed for *Klebsiella* spp., whose prevalence increased from 19.1% in 2019 to 23.6% in 2023, despite a lower proportion in 2021 (16.6%). In contrast, *Proteus* spp. showed no significant changes, accounting for 10.7% in 2019 and 9.1% in 2023, with only a mild decrease during 2020, 2021, and 2022. Over time, the prevalence of *Enterobacter* spp. declined, dropping from 3.2% in 2019 to 1.3% in 2023. The prevalence of *Citrobacter* spp. corresponded to 0.9% in 2019 and remained low throughout the study period, increasing slightly to 1.3% in 2023. At the same time, *Morganella* spp. was more frequently detected in 2019 (2.3%), decreased to 0.9% in 2023, and showed a temporary peak in 2022 (3.5%). It is noteworthy that no samples collected in 2019, 2020, or 2023 included any *Acinetobacter* or *Providencia* species. These pathogens were detected only once in 2022, each accounting for 0.5% of isolates. Overall, these findings highlight the persistent prevalence of *E. coli* and indicate a mild increasing trend *Klebsiella* and *Proteus* isolates over the study period (Figure 1).

### 2.4. Antimicrobial Resistance Characteristics

Analysis of the AMR profile revealed a heterogeneous distribution across the 890 isolates analysed. Most microorganisms exhibited multiple resistances: categories with four, five, and six resistances were the most common, with frequencies of 16.6%, 21.7%, and 18.1% respectively. A considerable proportion of isolates (15.7%) showed resistance to seven antimicrobials, while only a small fraction (less than 4%) displayed extreme resistance profiles involving ten or more antimicrobials. In summary, the highest percentage of isolates showed multi-resistance (98.4%), while in only 1.6%, one resistance was detected. As regards the number of microorganisms isolated per sample, in almost all cases (93.2%) a single pathogen was identified, while co-isolation of two or more species was relatively uncommon (6.8% overall) (Table 8).

**Table 8:** Distribution of the resistance per sample.

Resistance Profile	N (%)
***Number of resistances***	
1	14 (1.6)
2	16 (1.8)
3	66 (7.4)
4	148 (16.6)
5	193 (21.7)
6	161 (18.1)
7	140 (15.7)
8	87 (9.8)
9	36 (4.0)
10	17 (1.9)
11	7 (0.8)
12	5 (0.6)
***Multi-resistance***	
No	14 (1.6)
Yes	876 (98.4)
***Number of isolated*** ***microorganisms***	
1	830 (93.2)
2	53 (6.0)
3	7 (0.8)

Among all the pathogens identified in this study, we focused on the antimicrobial resistance profiles of the three most frequently isolated species: *E. coli*, *K. pneumoniae*, and *Proteus mirabilis* (*P. mirabilis*).

**Figure 2:**
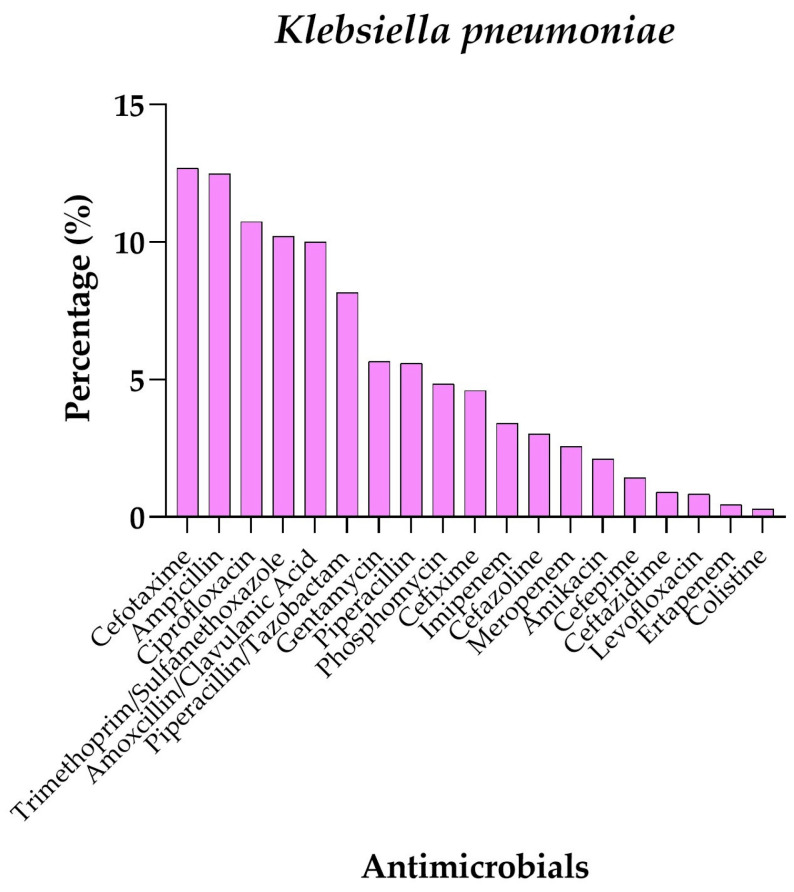
Percentage resistance profile of *Klebsiella pneumoniae* against different antimicrobials.

Considering *K. pneumoniae* (Figure 2), a high prevalence of resistance to antimicrobials such as Cefotaxime (12.7%), Ampicillin (12.5%), and Ciprofloxacin (10.7%) is evident. On the contrary, lower percentages of isolates showed resistance to Amikacin (2.1%) and Imipenem (3.4%). Interestingly, *K. pneumoniae* exhibited the lowest resistance rates to Ertapenem and Colistin, at 0.5% and 0.3%, respectively, suggesting a certain level of effectiveness of these antimicrobials against the pathogen.

**Figure 3:**
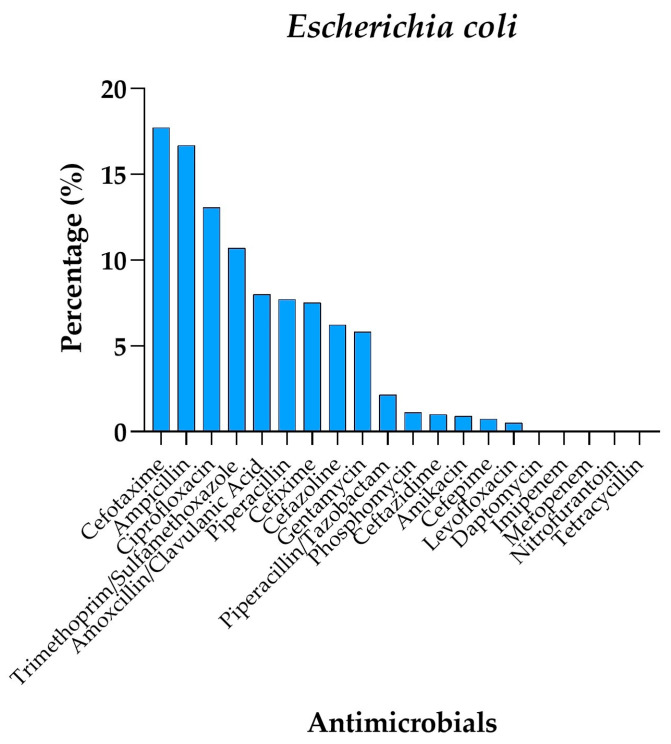
Percentage resistance profile of *Escherichia coli* isolates against different antimicrobials.

Regarding *E. coli* (Figure 3), the antimicrobials with the highest resistance rates were Cefotaxime (17.7%), Ampicillin (16.7%), and Ciprofloxacin (13.1%). Additionally, resistance to Ampicillin/Clavulanic Acid and Trimethoprim/Sulfamethoxazole was observed in 8% and 10.7% of isolates, respectively. In contrast, antibiotics such as Daptomycin, Meropenem, and Imipenem displayed very low resistance rates (0.03–0.03%), confirming their potential effectiveness against this pathogen.

**Figure 4:**
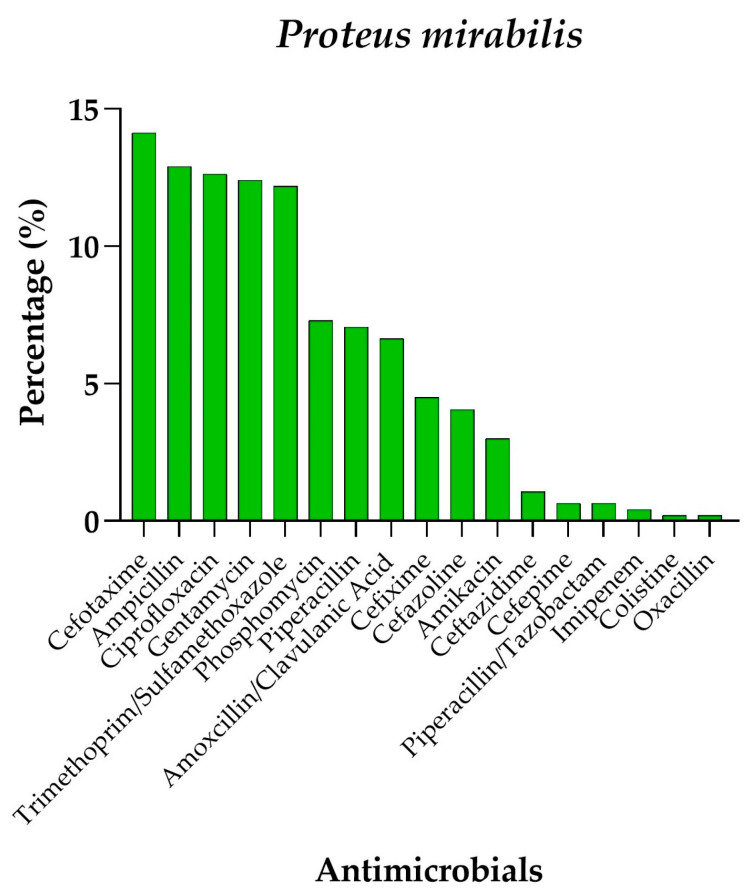
Percentage resistance profile of *Proteus mirabilis* isolates against different antimicrobials.

Finally, as evidenced by the analysis of *P. mirabilis*, antimicrobial resistance data revealed significant resistance to drugs such as Cefotaxime (14.1%), Ampicillin (12.9%), and Ciprofloxacin (12.6%), with values comparable to those observed for *E. coli*. As shown in the graph (Figure 4), Amikacin showed a relatively low level of resistance (3%), as did Piperacillin/Tazobactam (0.6%) and Imipenem (0.4%), suggesting that these antibiotics might be more effective against the pathogen compared to others.

**Figure 5:**
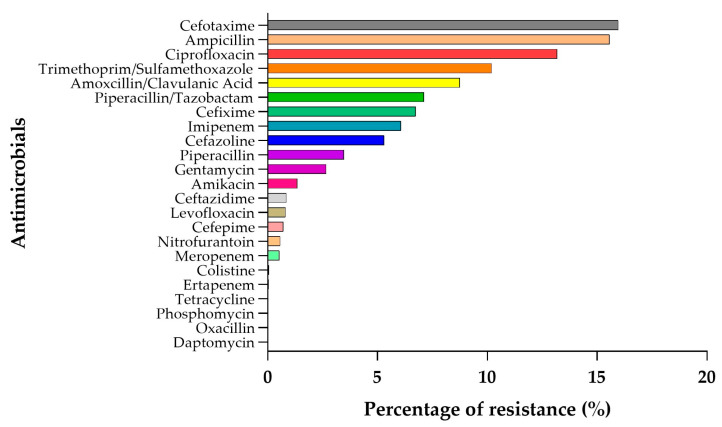
Distribution of antimicrobial resistance of all isolates over the whole study period (2019 to 2023).

In order to gain an overall view of the samples, the percentages of resistance of all clinical isolates against the tested antimicrobials were also analysed (Figure 5). As expected, the antimicrobials with the highest resistance rates correspond to those identified for the most prevalent species. Conversely, the agents demonstrating the greatest effectiveness were Tetracycline, Phosphomycin, Oxacillin, Ertapenem, Daptomycin, and Colistin.

**Figure 6:**
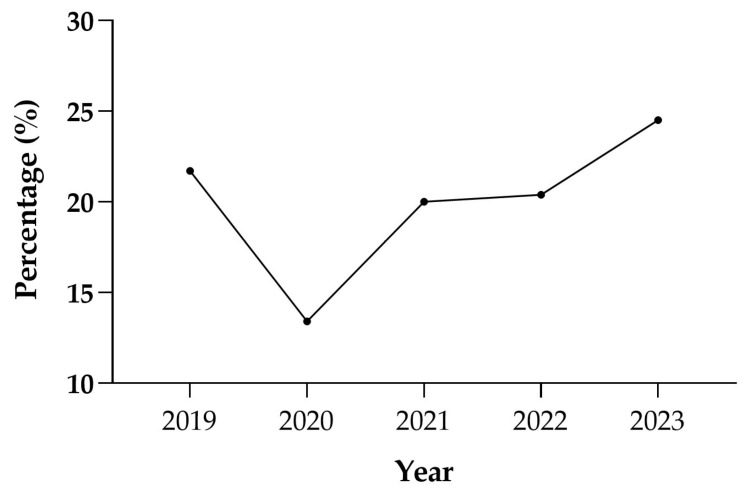
Percentage of resistance over time.

Lastly, to evaluate the trend in resistance over time, all the samples exhibiting at least one resistance were plotted according to the year of isolation (Figure 6). This approach provided a snapshot of the evolution of antimicrobial resistance over the years and showed that, with the exception of 2020 and slight decreases observed in 2021 and 2022, the frequency of resistance tended to increase in the final year compared to the initial one (*p* < 0.001).

## 3. Discussion

This retrospective study aims to describe the trend of AMR among Enterobacteriaceae isolated from hospitalised and non-hospitalised patients over a 5-year period (from 2019 to 2023). Samples were collected in Vasto (Chieti, Italy) and analysed at the Laboratory of Microbiology, Operative Unit of Clinical Pathology of “S. Pio” Hospital. The most representative samples were urine cultures (79.3%), suggesting a possible association with urinary tract infections, which are frequently related to the prolonged use of bladder catheters. Such devices represent a risk factor for nosocomial infections related to multi-resistant Enterobacteriaceae [32]. In these cases, biofilm formation on catheter materials plays a crucial role by facilitating bacterial adhesion, protecting microorganisms from the action of antimicrobials and contributing to the chronicity of the infection [33,34]. This issue represents a major challenge in the management of healthcare-related infections. Only 6.7% of the samples represented a condition of co-infection, while at least one resistance was detected in each sample. The number of resistances ranged from 1 to 12 and the final percentage of multi-resistance corresponded to 98.4%, while only 1.6% of samples showed only one resistance.

Our findings can contribute to understanding the growing phenomenon of antimicrobial resistance, which represents a serious threat with significant public health and economic implications [10]. Understanding the evolution of resistant pathogens is necessary to support the development of new treatment and surveillance strategies; thus, in 2024, the WHO released the latest update of the Bacterial Priority Pathogens List (BPPL). As in previous editions, antibiotic-resistant bacteria were classified into three priority groups—critical, high, and medium. Carbapenem- and third-generation cephalosporin-resistant Enterobacterales and carbapenem-resistant *A. baumannii* were listed in the critical group, followed by vancomycin-resistant *Enterococcus faecium* (*E. faecium*) categorised as “high” priority [35].

The data collected in this retrospective study clearly showed that the most frequently isolated species over the entire observation period were *E. coli* and *K. pneumoniae*. This finding is consistent with a briefing released by the Institute for Health Metrics and Evaluation (IHME), which reported that in 2019, in Italy, these two species were among the pathogens associated with the highest number of AMR-related deaths [36]. Furthermore, several studies have confirmed the concerning spread of *E. coli* and *K. pneumoniae* in Italy in the following years, with outbreaks of these drug-resistant bugs documented in regions such as Apulia [37] and Lombardia [38,39,40]. Numerous works have recently revealed the high frequency of isolation of these two bacterial species worldwide. In 2024, Budia-Silva et al. published an interesting study monitoring the spread of carbapenem-resistant *K. pneumoniae* in nine Southern European countries over a 3-year period, focusing on the distribution of different clonal lineages identified through genome analysis [41]. In a detailed review about ESBLs, Husna and colleagues provided data from the public and animal health sectors in Bangladesh, India, and Pakistan collected from 2015 to 2023 showing the highest prevalence of both *E. coli* and *K. pneumoniae* [42]. Similarly, in 2025, Singh et al. confirmed a comparable overview in their prospective observational study analysing samples from healthcare centres in the metropolitan area of Kerala, India [43]. These two species were also identified as the most prevalent isolates in Africa and Russia, according to results from clinical samples, as well as in Switzerland, where Aguilar-Bultet et al. identified presumptive ESBLs-producing Enterobacteriaceae from wastewater samples [44,45].

Another practical observation emerging from these investigations is that, despite the widespread prevalence of the same bacterial species, the resistance rate towards specific antimicrobial categories may vary considerably according to the geographical area. Developing countries, for example, are critical hotbeds for antibiotic-resistant bacteria because several factors, such as poverty, drug quality, and the lack of adequate surveillance, contribute to this phenomenon [42]. On the other hand, in developed countries, antimicrobial resistance is often linked to different drivers, including the indiscriminate use of antibiotics in health care and food production [46]. Furthermore, the sample size and additional ecological and environmental determinants should be taken into account, as each of them shapes the whole epidemiological scenario [47]. Focalising the attention on Southern Italy, a retrospective study of Barchitta et al., carried out over three-years in Sicily, demonstrated that *K. pneumoniae* isolates collected from hospitals showed an increase in resistance to third-generation cephalosporins, fluoroquinolones, and colistin during the study period; on the contrary, the resistance of *E. coli* isolates to the same antimicrobials declined [48]. As previously mentioned, variations in antimicrobial resistance depend on the microbial species, administered antimicrobials, and geographical area. In our study, considering all isolates collected over the 5-year period, the highest resistance rates were observed for Cefotaxime (16.0%), Ampicillin (15.6%), Ciprofloxacin (13.2%), and Trimethoprim/Sulfamethoxazole (10.2%). Specifically, among the Enterobacteriaceae family, resistance to third generation cephalosporins, penicillins, and fluoroquinolones is consistent with data reported in the 2023 annual surveillance report, published by the European Centre for Disease Prevention and Control (ECDC) which provides an overview of AMR in bloodstream infections across EU/EEA countries [49]. Similar results have been reported in Russia, where a 7-year study found that Ciprofloxacin and Ampicillin were the least effective antibiotics against Enterobacteriaceae isolates, with resistance rates of 52.3% and 80.8%, respectively [45]. A comparable trend was also observed by Zafer and colleagues who isolated *K. pneumoniae* and *E. coli* from cancer patients in Egypt: their study revealed high resistance rates to β-lactam and sulphonamide antimicrobials [50]. These studies also revealed an increasing prevalence in Colistin resistance, which has been also confirmed by many authors, and it is emerging as a serious concern [51,52,53]. On the contrary, in our work, most isolates showed susceptibility to this antibiotic, registering a resistance rate of only 0.06%, confirming a previously published report that demonstrated that aminoglycosides and Colistin are among the more efficacious drugs against Enterobacteriaceae [54]. While these findings may be partly limited by the sample size and differences in the experimental design across studies, they nonetheless reflect specific characteristics of the enrolled population.

The last aspect we focused on was the trend in antimicrobial resistance over the years, which clearly showed an increase in 2023 compared to 2019. It is worth noting that, even if it is not statistically significant, in 2020 there was a decline that did not align with the general progression. We speculate that this reduction could be attributed to the COVID-19 pandemic, which may have caused disruptions in the monitoring system. In fact, some authors have suggested that, due to the public health emergency, antimicrobial-resistance surveillance was deprioritised, and the laboratory resources for antimicrobial susceptibility testing were reduced, potentially leading to an underestimation of the resistance spread [55,56]. Furthermore, it should be underlined that another alarming occurrence during this period was the uncontrolled administration of antibiotics, especially in the early stages of the pandemic [55].

Taken together, all this evidence clearly underscores the value of observational studies, which show the evolving threat of antimicrobial-resistance and inform the design of more effective strategies.

## 4. Materials and Methods

### 4.1. Patients

In this study 890 patients were enrolled, 61.3% of female gender and 38.7% of male gender, aged between 1 and 99 years, resulting in a median age of 73 years (IQR = 66–85). Among them, 58.5% and 41.5% were hospitalised and non-hospitalised, respectively. The largest percentage of samples taken in hospitalised patients were 9.3% from geriatrics, 9.3% from internal medicine, and 4.2% from intensive care. Sample collection and analysis were carried out over a period of five years, from 1st January 2019 to 31st December 2023, at the Laboratory of Microbiology, Operative Unit of Clinical Pathology of “S. Pio” Hospital of Vasto, Chieti, Italy.

### 4.2. Sample Collection, Bacterial Species Identification, and Antimicrobial Susceptibility Pattern Determination

The biological samples were collected using standard procedures, depending on the sampling site. Biological samples were collected in the form of urine culture, urine from a paediatric bag, catheter urine, blood culture and intravascular catheter tips, vaginal swab, sputum, bronchial lavage, tracheobronchial aspirate, broncho-alveolar lavage, ear swab, secretions from wounds or abscesses, pus, biopsies of bedsores, skin swab, seminal fluid, and urethral swab. The majority of samples (79.3%) were obtained from urine cultures followed by hemocultures (8.4%) and skin swabs (4%). The remaining sampling sources varied between 2.5 and 0.1%. Urine samples were collected preferably in the morning, following thorough perineal hygiene by the patient, according to the standard procedures for midstream urine collection. The samples were then delivered to the Microbiology Laboratory of “San Pio” Hospital in Vasto either by the patient or by healthcare personnel. Upon arrival, the samples were processed immediately or stored at 4 °C for a maximum of 8 h. Alternatively, urine specimens collected in urine culture tubes containing boric acid as preservative (Urine Monovette® with Boric Acid, Sarstedt GmbH, Nümbrecht, Germany, DEU) were stored for up to 24 h, in accordance with the current guidelines for maintaining microbiological stability. Swab samples were transferred into Amies transport medium (MicroBiotech Maglie, Italy, ITA), which allows their storage until processing. Blood samples were collected and subsequently analysed using BacT/ALERT 3D (BioMérieux, Marcy-l’Étoile, France, FRA) that monitors the incubated samples every 10 min to determine the microbial growth curve. After collection, samples were plated on selective agar plates, such as Columbia blood agar, MacConkey agar, Schaedler Vancomycin Selective agar (BioMérieux, Marcy-l’Étoile, France, FRA) and Sven Gard agar (Bio-Rad, Hercules, CA, USA), and incubated in aerobic or anaerobic conditions for 18–24 h or 48 h. The microorganisms were identified considering the colonies’ morphology, the Gram staining, and by the MicroScan WalkAway 40 Plus system (Beckman Coulter, Brea, CA, USA), an automated antimicrobial susceptibility testing system, which guarantees the identification of microbial species and their antimicrobial resistance pattern. The panels used in this study are the Pos Breakpoint combo Panel type 32 (PBC 32) and the Neg Breakpoint Combo Panel 81, 82, and 83 (NBC 81, 82, and 83). PBC 32 was used for both the identification of aerobic and optional fast-growing Gram-positive cocci and some demanding aerobic Gram-positive cocci. In addition, this panel allows determination of the susceptibility to antimicrobial agents. NBC 81, 82, and 83 were used for the identification of Gram-negative species. The results were interpreted according to EUCAST, CLSI M100-S19, and CASFM breakpoints [57,58,59].

### 4.3. Statistical Analysis

The statistical analysis was carried out as described in the previously published scientific article [60]. In particular, categorical variables were reported as absolute and percentage frequencies, while continuous variables were described by median and interquartile interval (IQR). The association between groups was tested with the chi-square test. The Mantel–Haenszel test was used to determine whether there was a statistically significant linear trend in the proportion of infections caused by the most frequently isolated microorganisms during the study period. A value of *p* ≤ 0.05 was indicative of statistical significance. Statistical analyses were performed using IBM^®^ SPSS Statistics version 20.0 (SPSS Inc., Chicago, IL, USA) and GraphPad Prism version 7 (GraphPad Software, La Jolla, CA, USA).

### 4.4. Ethical Considerations

The study was performed according to the Italian law on privacy (Art.20-21 DL 196/2003), published in the Official Gazette, n. 190, on 14 August 2004. The data were encrypted prior to the analysis at the Hospital S. Pio of Vasto, Chieti (Italy), where a unique identifying code was assigned to each patient. The unique code eliminated the possibility of identifying the patients’ identities. According to Italian law, the use of administrative data does not require any written informed consent from patients. The present study did not directly involve human patients.

## 5. Conclusions

The aim of the present retrospective study was to take a snapshot of the current situation concerning the antimicrobial susceptibility pattern of the Enterobacteriaceae family in an area of our region. The analysis of the drug resistance versus different antimicrobials regardless of class membership could contribute to a more informed and targeted choice of antimicrobial drugs to be administered to patients, in particular if conditions arise in which it is not possible to carry out antimicrobial susceptibility tests. The data obtained demonstrated that there are fourteen effective antimicrobials for the treatment of the Enterobacteriaceae-associated infections, with a percentage of resistance ≤ 5%, among the ones commonly tested. Based on our findings, it is worth noting that antibiotics belonging to the same class or generation displayed a different percentage of resistance versus Enterobacteriaceae isolates, as for example Cefotaxime, Cefixime, and Ceftazidime. The results of this study represent an important contribution to the optimisation of antimicrobial therapy, providing clinicians with useful tools for selecting more effective treatments, both in the management of Enterobacteriaceae infections and in preventing the spread of multi-resistant strains. The adoption of therapeutic regimens based on local antimicrobial sensitivity data can promote a more rational and targeted use of antimicrobials, contributing significantly to the reduction in the use of less effective drugs and, as a result, to the containment of AMR. Combating the spread of multi-resistant microorganisms requires an integrated approach, including constant microbiological surveillance, periodic updating of therapeutic guidelines, and the adoption of antimicrobial stewardship policies. In line with the recommendations of the World Health Organization, these interventions are essential to tackle one of the most serious threats to global public health today.

## Figures and Tables

**Table 1:** Sample characteristics: year, gender, patients’ group, and co-infection.

Characteristics	N (%)
Samples	
2019	193 (21.7)
2020	119 (13.4)
2021	178 (20.0)
2022	182 (20.4)
2023	218 (24.5)
Gender	
F	546 (61.3)
M	344 (38.7)
Patients group	
Hospitalised	520 (58.5)
Non-hospitalised	370 (41.5)
Co-infection	
No	830 (93.2)
Yes	60 (6.8)

**Table 2:** Distribution of samples collected from hospitalised and non-hospitalised patients in different hospital departments.

Ward	N (%)
***Hospitalised***	
Internal Medicine	83 (9.3)
Geriatrics	83 (9.3)
Infectious Diseases	56 (6.4)
Intensive Care Unit	37 (4.2)
Surgery	23 (2.6)
Neurology	18 (2.0)
Urology	14 (1.6)
Cardiac Intensive Care Unit	9 (1.0)
Dialysis	9 (1.0)
Paediatrics	8 (0.9)
Oncology	7 (0.8)
Nephrology	6 (0.7)
Orthopaedics	5 (0.6)
Gynaecology	3 (0.3)
Long-Term Care	3 (0.3)
Cardiology	2 (0.2)
Obstetrics	2 (0.2)
Psychiatry	1 (0.1)
Pulmonology	1 (0.1)
***Non-hospitalised***	
Outpatients	288 (32.4)
Community Health Centre	164 (18.4)
ADI (Home Assistance)	67 (7.5)
Correctional Facility	1 (0.1)

**Table 3:** Distribution of samples based on specimen type.

Type of Specimen	N (%)
Urine	705 (79.3)
Blood	75 (8.4)
Skin Swab	36 (4.0)
Vaginal Swab	22 (2.5)
Sputum	14 (1.6)
Peritoneal Fluid	13 (1.5)
Tracheal Aspirate	8 (0.9)
Pus	6 (0.7)
Bronchoalveolar Lavage	2 (0.2)
Rectal Swab	2 (0.2)
Seminal Fluid	2 (0.2)
Cervical Swab	1 (0.1)
Joint Fluid	1 (0.1)
Nasal Swab	1 (0.1)
Umbilical Swab	1 (0.1)
Urethral Swab	1 (0.1)

**Table 4:** Distribution of the isolated microorganisms.

Microorganisms	N (%)
*Escherichia coli*	604 (63.2)
*Klebsiella* spp.	209 (21.9)
*Proteus* spp.	84 (8.8)
*Enterobacter* spp.	20 (2.1)
*Morganella morganii*	17 (1.8)
*Citrobacter* spp.	12 (1.2)
*Serratia marcescens*	8 (0.8)
*Acinetobacter baumannii*	1 (0.1)
*Providencia stuartii*	1 (0.1)

**Table 5:** Distribution of multi-resistance by age group (*p* = 0.199).

Age (Years)	Multi-Resistance	
	Yes N (%)	No N (%)
≤65	211 (97.7)	5 (2.3)
66–75	202 (98.1)	4 (1.9)
76–85	257 (98.8)	3 (1.2)
>85	206 (99.0)	2 (1.0)

**Table 6:** Distribution of multi-resistance by gender group (*p* = 0.386).

Gender	Multi-Resistance	
	Yes N (%)	No N (%)
F	539 (61.5)	7 (50.0)
M	337 (38.5)	7 (50.0)

**Table 7:** Distribution of multi-resistance by years (*p* = 0.490).

Years	Multi-Resistance	
	Yes N (%)	No N (%)
2019	191 (21.8)	2 (14.3)
2020	115 (13.1)	4 (28.6)
2021	175 (20.0)	3 (21.4)
2022	178 (20.3)	4 (28.6)
2023	217 (24.8)	1 (7.1)

## Data Availability

The datasets generated and analysed in the current study are available from the corresponding author on reasonable request.
